# Black Box Warning: Large Language Models and the Future of Infectious Diseases Consultation

**DOI:** 10.1093/cid/ciad633

**Published:** 2023-11-16

**Authors:** Ilan S Schwartz, Katherine E Link, Roxana Daneshjou, Nicolás Cortés-Penfield

**Affiliations:** Division of Infectious Diseases, Department of Medicine, Duke University School of Medicine, Durham, North Carolina, USA; Department of Medical Education, Icahn School of Medicine at Mount Sinai, NewYork, New York, USA; Healthcare & Life Sciences Division, Hugging Face, Brooklyn, NewYork, USA; Department of Dermatology, Stanford School of Medicine, Stanford, California, USA; Department of Biomedical Data Science, Stanford School of Medicine, Stanford, California, USA; Division of Infectious Diseases, University of Nebraska Medical Center, Omaha, Nebraska, USA

**Keywords:** artificial intelligence, ChatGPT, chatbot, natural language processing, workforce

## Abstract

Large language models (LLMs) are artificial intelligence systems trained by deep learning algorithms to process natural language and generate text responses to user prompts. Some approach physician performance on a range of medical challenges, leading some proponents to advocate for their potential use in clinical consultation and prompting some consternation about the future of cognitive specialties. However, LLMs currently have limitations that preclude safe clinical deployment in performing specialist consultations, including frequent confabulations, lack of contextual awareness crucial for nuanced diagnostic and treatment plans, inscrutable and unexplainable training data and methods, and propensity to recapitulate biases. Nonetheless, considering the rapid improvement in this technology, growing calls for clinical integration, and healthcare systems that chronically undervalue cognitive specialties, it is critical that infectious diseases clinicians engage with LLMs to enable informed advocacy for how they should—and shouldn’t—be used to augment specialist care.

## Teaser

Large language models (LLMs), advanced artificial intelligence systems capable of generating natural language, could revolutionize healthcare, including current models of specialist consultation. Infectious diseases clinicians must urgently engage with and understand limitations of LLMs to advocate for their responsible integration.

## Graphical abstract

**Graphical Abstract:**
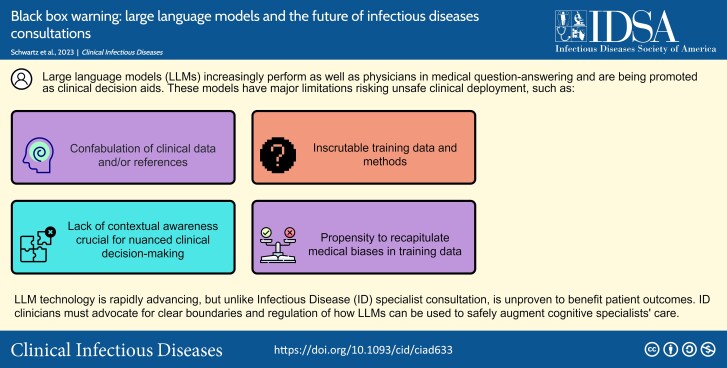
This graphical abstract is also available at Tidbit: https://tidbitapp.io/tidbits/black-box-warning-large-language-models-and-clinical-consultation-in-infectious-disease

**(See the Editorial Commentary by Kanjilal on pages 867–9.)**

In his 1970 futuristic essay, “Medicine and the Machine,” Dr. William B. Schwartz [1] predicted,

“[I]n the not too distant future the physician and the computer will engage in frequent dialogue, the computer … alerting the physician to the most probable diagnoses and suggesting the appropriate, safest course of action.… Because the computer has a large potential role as tomorrow's “consultant”, physicians engaged in consulting activities … will be much less in demand if interactive programs… can give prompt and expert counsel on the full range of problems encountered in clinical medicine.”

Computers' promise as clinical consultants has long gone unfulfilled. Recently, however, advances in large language models (LLMs)—artificial intelligence (AI) systems trained to process human language and predict text responses—have led to speculation that they may replace or augment consultant physicians in some functions. For example, Lee and colleagues suggested that Generative Pretrained Transformer (GPT)-4 (OpenAI) can be used for “curbside consults” [2]. While LLMs will surely improve administrative tasks that now inundate clinicians, their potential to safely inform clinical management decisions is currently diminished by several important limitations, including a propensity to authoritatively dispense inaccurate information (the effects of which are compounded by a human tendency to overlook machine errors), lack of transparency and output reproducibility, and potential for bias. Consequently, premature and unscrupulous deployment of LLMs in clinical settings could lead to serious patient harm. In this Viewpoint, we introduce infectious diseases (ID) consultants to LLMs, explore their strengths and limitations pertaining to clinical use, and call for engagement and advocacy for their safe use.

## HOW LARGE LANGUAGE MODELS WORK

Large language models are machine-learning algorithms trained to recognize and predict linguistic patterns (see Table 1 for a glossary of relevant terms). Most of this learning is self-supervised and gleaned from internet texts, including websites, social media forums, and open-access code, books, and articles. Many LLMs subsequently undergo supervised training (eg, reinforcement learning with human feedback [RLHF]), during which the model learns from human ratings of its responses.

**Table 1.** Glossary of Relevant Machine-Learning and Natural Language–Processing Terms

Glossary	Meaning
Artificial intelligence (AI)	Machine programs trained to do cognitive tasks typically requiring a human.
Generative AI	A subset of AI that involves the generation of new data (eg, text, images, music, speech, or video) based on input prompts.
Machine learning (ML)	A process of training machines to recognize patterns. Deep learning is a type of ML that uses complex layers of hidden data; supervised ML uses labeled data to train a model, whereas unsupervised learning aims to find patterns in unlabeled data.
Large language models (LLMs)	A type of generative AI trained on large corpora of texts to process natural language. Modern LLMs demonstrate additional functionalities like writing code, interpreting and translating images to and from text, and even acting autonomously to complete complex, long-term goals.
Generative Pretrained Transformer (GPT)	A type of LLM that includes some of the largest and most capable models circa early 2023, powering the popular chatbot ChatGPT (OpenAI).
Confabulation	More commonly referred to in the literature as a “hallucination.” A common and undesirable feature of LLMs, in which the model produces fluent but factually incorrect or off-topic output.
Automation bias	A cognitive bias toward unduly trusting or preferring the output of automated systems while ignoring contradictory information.

General-purpose LLMs like OpenAI's GPT-3 and GPT-4, Google's BERT, and Meta's LLaMA—sometimes called foundation models—have been trained on massive quantities of text without specific domain (eg, biomedical) training [3]. Alternatively, some LLMs, like Microsoft Research's BioGPT [4] and Google's Med-PaLM and Med-PaLM 2 [5], have been trained or fine-tuned on biomedical texts to develop medical-domain functionality. Both types of LLMs can answer medical questions with information acquired from their training data, evident in several such models (GPT-3.5, GPT-4, Med-PaLM, Med-PaLM 2) achieving passing scores on simulated versions of the US Medical Licensing Examination [5, 6].

Despite their skill at processing natural language to generate compelling, seemingly insightful texts, it is worth remembering that LLMs operate by recognizing linguistic patterns and predicting the words and phrases most likely to follow based purely on statistical computations. Bender and colleagues [7] aptly described LLMs as “stochastic parrots,” creatures that function by “haphazardly stitching together sequences of linguistic forms … according to probabilistic information about how [words] combine, but without any reference to meaning”. Large language model compute language form, but not its meaning, and easily generate medical text as persuasive as it is wrong.

## LIMITATIONS OF CURRENT LARGE LANGUAGE MODELS RELEVANT TO CLINICAL APPLICATIONS

### Confabulations

Because LLMs are trained to generate text based on prior contextual clues and stochasticity rather than factual correctness, they are prone to what AI specialists anthropomorphically term “hallucinations,” but which clinicians will better recognize as “confabulations”: coherent and confident but incorrect statements. Confabulations can be simple, elaborate, or even nonsensical, but the authoritative style in which they are presented can confound their detection.

Confabulations can also be dangerous. For example, when we asked GPT-3.5 to write a management plan for a patient with human immunodeficiency virus (HIV)–associated cryptococcal meningitis, it produced a detailed and structured list of suggestions (Box 1). While the plan may appear robust and appropriate to nonspecialists, it contains critical errors. The recommendations to immediately initiate antiretroviral therapy (ART)—which is proven by a randomized clinical trial to increase mortality compared with deferred ART initiation in people with HIV who are diagnosed with cryptococcal meningitis [8]—and to control increased intracranial pressure medically rather than by cerebrospinal fluid removal are in dire contradiction to clinical practice guidelines, and could lead to serious patient harm [9]. In another example (Supplementary Box 1), GPT-3.5 incorrectly asserted (among other serious medication errors) that histoplasmosis can be treated with echinocandins, confabulating a case series as supporting evidence.

**Box 1.** Example of critical confabulations in a management plan for cryptococcal meningitis, written by ChatGPT (GPT-3.5) and missed on “cross-checking” by ChatGPT (GPT-4). If unrecognized by the user, 2 of these recommendations could lead to serious patient harm.

Clinician	“Please write a detailed management plan for a patient with cryptococcal meningitis…”
ChatGPT (GPT-3.5)	“… Here is a detailed management plan for a patient with cryptococcal meningitis: 1. Diagnosis: The diagnosis of cryptococcal meningitis is confirmed by analyzing cerebrospinal fluid (CSF) obtained by lumbar puncture. The presence of *Cryptococcus neoformans* antigen or the fungus itself on CSF examination confirms the diagnosis. 2. Antifungal therapy: The cornerstone of management is antifungal therapy, which aims to eliminate the fungus from the CSF and prevent recurrence. The induction phase involves the use of amphotericin B, in combination with flucytosine, for 2 weeks. Subsequently, patients are transitioned to fluconazole, given at high doses for an additional 8–10 weeks, followed by maintenance therapy with lower doses for up to 1 year… 3. Management of raised intracranial pressure (ICP): … In patients with elevated ICP, prompt management is essential, including administration of acetazolamide, mannitol or hypertonic saline, and consideration of CSF drainage if necessary. 4. Immune reconstitution: Immune reconstitution therapy with antiretroviral therapy (ART) should be initiated in patients with human immunodeficiency virus (HIV)/AIDS as soon as possible… ART should be initiated within 2 weeks of antifungal therapy initiation, provided there are no contraindications …”
Clinician	“Please assess this management plan for cryptococcal meningitis:” [above plan inserted]
ChatGPT (GPT-4)	“This management plan for cryptococcal meningitis appears comprehensive and well-informed, adhering to clinical practice guidelines and recommendations … “
Clinician	“When should ART be started? please provide references”
GPT-4	“According to the guidelines for the diagnosis, prevention, and management of cryptococcal disease in HIV-infected adults, adolescents, and children, ART should be initiated within 2 weeks of starting antifungal therapy for cryptococcal meningitis, provided there are no contraindications. This recommendation is based on evidence suggesting that earlier initiation of ART improves immune function and reduces the risk of mortality and recurrence of cryptococcal meningitis. Reference: World Health Organization. Guidelines for the diagnosis, prevention and management of cryptococcal disease in HIV-infected adults, adolescents and children. Geneva, Switzerland: World Health Organization; 2018. Available at: https://www.who.int/hiv/pub/guidelines/cryptococcal-disease/en/. It is important to closely monitor patients for signs of immune reconstitution inflammatory syndrome (IRIS) when initiating ART, as this can lead to a paradoxical worsening of symptoms. Management of IRIS may require corticosteroids and other immunomodulatory therapies.”
Clinician	“What about the RCT by Boulware et al in NEJM 2010 that showed higher mortality when ART was started within 2 weeks?”
GPT-4	“You are right to bring up the study by Boulware et al., published in the *New England Journal of Medicine* (NEJM) in 2010. In this randomized controlled trial (RCT), the researchers found that starting ART within 2 weeks of initiating treatment for cryptococcal meningitis was associated with higher mortality, compared to starting ART after 2 weeks. Reference: Boulware DR, Meya DB, Muzoora C, et al. Timing of antiretroviral therapy after diagnosis of cryptococcal meningitis. N Engl J Med. 2014;370(26):2487–2498. doi:10.1056/NEJMoa1312884 …”
Comments	Two recommendations are dangerously wrong. Firstly, the suggestion to immediately start antiretroviral therapy is grievous: Boulware et al found higher mortality in patients with HIV-associated cryptococcal meningitis (CM) who were randomized to early (1–2 weeks) vs delayed (≥5 weeks) ART initiation relative to commencing antifungals. Secondly, increased cranial pressure is a major cause of mortality in CM, and must be lowered with CSF removal or diversion, not acetazolamide, mannitol or hypertonic saline. Note the fluent and authoritative nature of the output, including the errors, which can easily lead to being overlooked by non-experts, with potentially deadly consequences. In another chat, ChatGPT (GPT-4) verifies these recommendations, and—when pressed about the early ART suggestion—falsely cites as evidence the WHO practice guidelines (which strongly recommend against this practice). The chatbot can be coaxed to the correct answer. Note also how ChatGPT (GPT-4) mimics the user's error (Boulware et al paper was not published in 2010) despite “knowing” better (correctly cited in the chatbot's reference). This chat has been truncated for brevity, as indicated by the ellipses. The complete unabridged transcript of the chat is found in Supplementary Box 2.

Large language model errors are not always this egregious, but they occur frequently enough to warrant serious pause about the role of LLMs in clinical tasks. For example, only 13.7% of references GPT-3.5 produced when answering radiology questions were both authentic and relevant [10]. Johnson et al [11] tested GPT-3.5 with 284 physician-provided questions and judged only 39.4% of responses to be completely accurate, with 8.3% “completely inaccurate.” When Dash and colleagues [12] asked 12 physicians to evaluate GPT-3.5’s and GPT-4’s responses to 66 clinical scenarios, they found that one-third of answers from each model were flagged by at least 1 physician to be potentially harmful. Howard et al [13] tested GPT-3.5 against plausible ID “curbside consults” and found that “dangerous advice was repeatedly given despite prompting,” concluding that ChatGPT suffered from “deficits in situational awareness, inference, and consistency… [that] could endanger patient safety”. Sarink and colleagues [14] tested GPT-3.5 with actual ID cases and compared advice with that given by consultants, finding that even correct advice was often supported by misrepresented evidence. They concluded that “[t]he air of confidence in which ChatGPT occasionally presents factually incorrect responses … might put patients at risk”.

**Figure 1.**
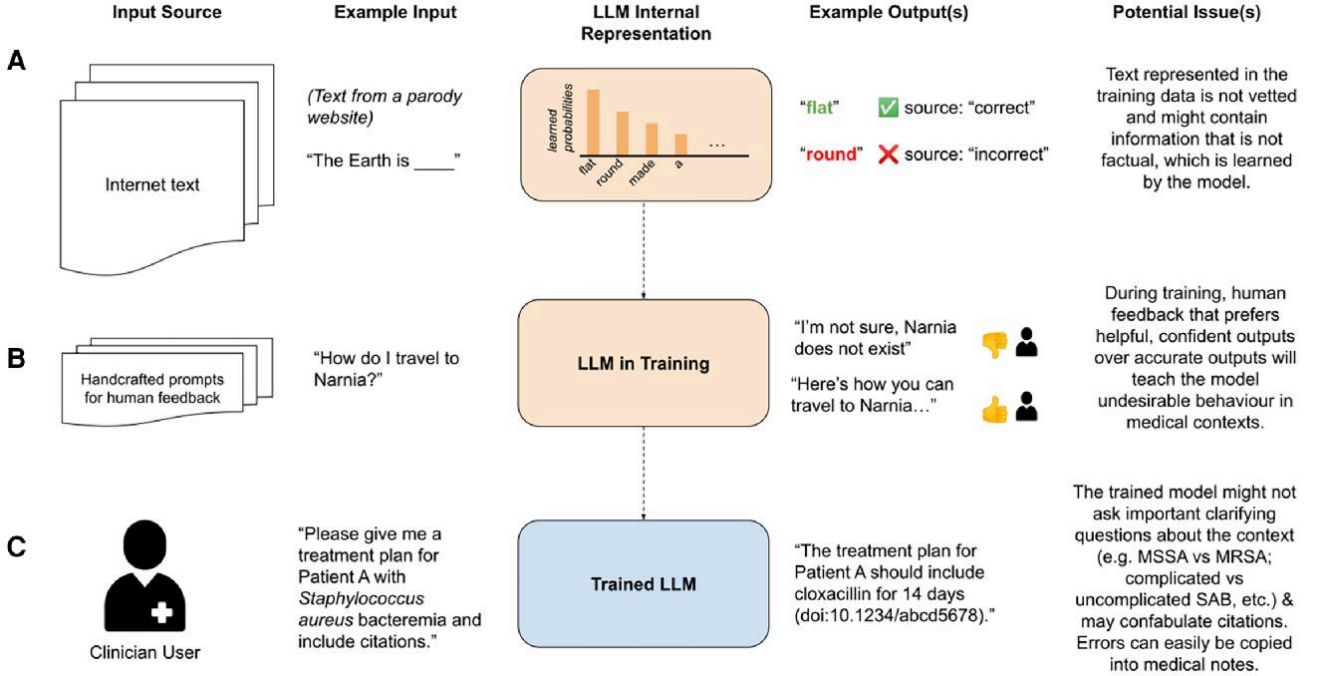
Overview of different steps during the training and deployment of LLMs. *A*, LLMs are first pretrained on massive amounts of text collected from the internet. Text represented in these training data might not be vetted and could contain factual errors, which could then be represented in the model's learned probabilities of tokens. *B*, After pretraining, models are then trained using supervised learning procedures, including a portion of training that uses human feedback to train a reward model that assigns ratings to model outputs. Since this reward model is trained by humans, who prefer certain qualities of outputs (such as helpfulness and confidence), this training procedure could lead to models with outputs that might be undesirable in domains where other qualities are desired (such as displaying uncertainty when the answer is not known). *C*, Trained models deployed in medical settings might be given prompts by clinician users without full clinical context, which could lead to the output of incorrect medical information that could affect clinical care if not properly vetted. Models also frequently are not able to produce accurate citations for studies. Both errors could be easily copied into a patient’s medical record. Abbreviations: LLM, large language model; MRSA, methicillin-resistant *Staphylococcus aureus*; MSSA, methicillin-susceptible *Staphylococcus aureus*; SAB, *Staphylococcus aureus* bacteremia.

The reasons that a specific confabulation occurs are obscured by the inability to examine an LLM's training data or decipher its computational machinations [15]. However, general reasons why they occur include inaccuracies or conflicting information in training data, lack of contextual awareness, incorrect inference of the user's intent based on ambiguity in the prompt, or over-optimization for language fluency at the cost of factual accuracy. Figure 1 illustrates several ways that inaccuracies can unintentionally be incorporated into the LLM's outputs during training or in deployment.

Large language models are also prone to errors caused by “falsehood mimicry”: improvising rather than confronting a falsehood in a prompt [16]; examples are shown in Box 1 and Supplementary Box 1. Falsehood mimicry is especially problematic because a compliant chatbot may not challenge inappropriate requests or assumptions from users. For example, when Howard et al [13] asked ChatGPT for antimicrobial advice for several hypothetical situations, it failed to challenge the request for discharge oral antibiotics for “*Streptococcus aureus*” bacteremia or for a patient in profound hypoxic respiratory failure and headed toward intubation.

There are several approaches being evaluated to mitigate confabulations, and their success will ultimately determine the viability of LLMs in clinical medicine. One approach is to use RLHF to correct errors and steer the LLM towards more desired output [17]; another is to ground the LLM's responses in text from reputable sources that has been converted into information-dense word embeddings [18]. Confabulation can also be influenced by the “temperature,” a model parameter reflecting the randomness of an output [eg, controlling whether it always selects the next most probable word or whether it can select from words with lower probabilities, yielding more unpredictable but potentially creative output). Large language models deployed specifically for healthcare could be adjusted to favor accuracy over creativity, but at the cost of lower functionality for tasks that require generation of new ideas—for example, “brainstorming” differential diagnoses.

Microsoft's Lee and colleagues [2] conceded that inaccurate outputs limited LLMs in healthcare but argued that GPT-4 could be used to cross-check LLM-generated texts. However, this is not only time-consuming, but ineffective (eg, Box 1 and Supplementary Box 1 wherein GPT-4 fails to detect the serious medical errors by GPT-3.5).

### Automation Bias

Confabulations are easily overlooked due to users' tendency toward deference to automated decision aids, a phenomenon termed “automation bias” [19]. People naturally take the cognitive path of least resistance [20], which frequently means accepting automated systems' output at face value. In the aviation industry, autopiloting systems failed to eliminate crashes partly because of human complacency and impaired decision making fostered by imperfect automated guidance, leading Skitka et al [21] to argue, “The presence of automated cues appears to diminish the likelihood that decision makers will either put forth the cognitive effort to seek out other diagnostic information or process all available information in cognitively complex ways.”

Automated bias is well documented in healthcare [22]. In one study, radiologists misclassified mammograms when suggested by a purported AI program [23]; in another, clinicians accepted automated interpretation of electrocardiograms with incorrect diagnoses of atrial fibrillation in one-quarter of cases, leading to unnecessary antiarrhythmics and anticoagulation in 10% [24]; in yet another, erroneous alerts from clinical decision-support systems during electronic prescribing led to an overall increase in prescribing errors [25].

We anticipate that automation bias will lead clinical decision-support LLMs to produce both errors of commission (eg, ART started too early in a patient with HIV-associated cryptococcal meningitis) or omission (eg, failure to ask about allergies or to recommend removal of a central venous catheter in a patient with *Candida* bloodstream infection). We also anticipate that LLM recommendations will be less likely to be challenged when they align with what users want to hear (eg, complying with a request for an oral antibiotic recommendation to facilitate inappropriately early discharge, or inappropriately clearing a patient to receive solid-organ transplantation or device implantation during an infection).

### Other Concerns

There are other attributes of current LLMs that limit their suitability for informing clinical management. For one, LLMs lack contextual awareness, which is particularly critical in ID, where diagnostic and therapeutic approaches must consider local epidemiology, antimicrobial resistance patterns, assay and drug availability, and other factors.

For another, current LLMs is their lack of transparency and “explainability” (the ability to understand how a specific machine works and how a particular output was derived from an input) [15, 26, 27]. These are “black box” models: their training data are often obscured or undocumented, and their methods opaque [7, 27]. The most advanced LLMs use deep neural networks with billions of parameters, precluding meaningful explainability in terms humans can understand [15]. Moreover, the details of trained models are frequently shielded behind corporate firewalls, including application programming interfaces (APIs) or chatbot user interfaces [28]. Lack of explainability can erode patient and physician trust [15, 26], and precludes the inspection, reflection, and learning required for safety and quality improvement [29]. Lack of explainability also makes LLMs vulnerable to cybersecurity threats that are difficult to detect [30]. For these reasons, some experts argue that black box models should never be used for high-stakes decisions [29].

Yet another concern is that AI systems can reinforce systemic biases in their training data, leading to discrimination and harm to marginalized groups [31, 32]. For example, an AI algorithm widely used by hospitals and insurers in the US systematically prioritized White patients over Black patients for healthcare resources [33]. LLMs also can recapitulate the biases in their training data [7], and may worsen existing inequalities in healthcare, especially if algorithmic judgements are erroneously presumed objective.

The AI tools marketed for use in clinical practice must be approved by the Food and Drug Administration (FDA), requiring rigorous validation like any other diagnostic or therapeutic device, although the FDA has yet to reveal its approach to regulating LLMs in healthcare [34]. However, the reference materials with which clinicians confer are not regulated.

Notably, legal scholars have concluded that physicians following AI recommendations inconsistent with standards of care can likely be held liable for patient harm [35]. Conducting reproducible studies validating outputs of models available through chatbot interfaces such as ChatGPT is also difficult because companies continuously update the models without notice [36] as well as the inability to control settings that dictate whether the outputs of the model are deterministic (ie, always producing the same output in response to the same prompt).

## POTENTIAL IMPACTS OF LARGE LANGUAGE MODELS ON THE INFECTIOUS DISEASES SPECIALIST WORKFORCE

### Could Large Language Models Help Narrow Healthcare Access Inequalities?

Infectious diseases specialists are an endangered species [37]. In 2017, 79.5% of US counties lacked a single ID physician, and 208 million people lived in counties with no or fewer than average ID physicians [38]. In 2022, the majority (54%) of US adult clinical ID training programs had unfilled fellowship positions during the match [37]. We anticipate arguments that LLMs should be employed to fill the gap between the supply and demand for ID expertise and narrow disparities in access in underserved regions. However, dispatching consultative LLMs in lieu of ID clinicians may worsen health inequalities by excusing suboptimal care of vulnerable patients (eg, by avoiding transfers to tertiary centers). Infectious diseases consultations improve outcomes in several serious infections; the same cannot be said about any LLM. Nevertheless, LLMs might bolster the ID workforce by reducing the administrative burdens associated with documentation, billing, and fielding routine patient questions around the clock, which contribute to physician burnout [39].

### Do Large Language Models Pose an Existential Threat to Cognitive Specialties like Infectious Diseases?

Although current LLMs cannot replace specialist consultants, it is critical to recognize that they represent the beginning, not the limit, of LLMs' rapidly advancing capabilities including for clinical consultation. For example, Med-PaLM 2, Google's second-generation, medical-domain fine-tuned LLM, substantially outperformed GPT-3.5 in medical questions, and largely outperformed physicians in answering long-form medical questions as judged by a panel of blinded physicians [5].

Infectious diseases physicians are undervalued in the US healthcare system, evident by reimbursement rates near the lowest among all physicians [37]. Unlike many other medical specialties, ID specialists have no unique jurisdiction over medical procedures or exclusive prescribing rights to specialized medications. Indeed, increasing adoption of ID telehealth models suggests that administrators and payors consider the in-person component of ID consultations dispensable. For these reasons, Schwartz's prediction of the effect of computer “consultants” on the economic demand for their human counterparts [1] seems prescient: when AI “clinical co-pilots” emerge, payors and hospital executives may wonder whether ID physicians and other cognitive specialists are needed at all.

### Use Cases for Large Language Models in Healthcare

Current LLMs might be well suited for administrative tasks that require standardized and predictable forms of writing grounded in accurate information provided by the user. In healthcare, these may include drafting documents (eg, pre-authorization letters) [40] and synthesizing texts (eg, from the electronic medical record for a discharge summary) [41]. It should be noted, however, that current publicly available LLMs (eg ChatGPT) may harvest user input for further training, and entering protected patient information into these systems likely breaches confidentiality. Large language model chatbots have shown promise in responding to simulated patient queries with empathy [42], although the accuracy of these responses has not been validated. Even here, caution and extensive validation are required to prevent catastrophic harm. For example, in 2020, a medical chatbot powered by GPT-3 and tasked by researchers with providing emotional support instead encouraged a mock patient to kill themselves [43]. In 2023, an AI chatbot deployed by the National Eating Disorder Association encouraged harmful dieting practices to people with histories of disordered eating [44]. Although designed as a rule-based chatbot [45], the late-stage integration of AI into the chatbot led to circumvention of safety guardrails [44–46].

## SHAPING THE FUTURE: A CALL TO ARMS

Given the rapid advancement of LLMs and the potential harms of their unscrupulous clinical deployment, it is urgent that ID clinicians participate in the development and oversight of these products. The development and inclusion of education on AI in both training programs as well as continuing medical education would provide a basis for general understanding around AI systems, including their strengths and weaknesses. This education would also provide ID clinicians with the language and technical knowledge necessary to engage with industry and academic partners to provide clinical guidance on LLM research and development. Guidance from ID clinicians could include the identification of problems suitable for LLM systems, the development of training and evaluation datasets for LLM research, as well as the design and management of prospective clinical trials to evaluate the efficacy and risks of LLMs in clinical settings. In addition, ID clinicians can participate in advocacy through regulatory agencies and professional societies for the safe, patient-centered development of LLM technology.

## CONCLUSION

Despite the hype, existing LLMs are not safe for use in clinical consultation. Seeking an LLM's clinical advice outside the user's medical expertise is particularly dangerous: the combination of LLMs' confabulations, our natural tendency toward cognitive complacency when interacting with automated systems, and the complexity and frailty of real patients portends disaster. Moreover, LLMs lack contextual awareness, are opaque, and may perpetuate bias. Their use in medical decision making should be curtailed until improved iterations undergo rigorous clinical validation.

Nevertheless, the field of AI is rapidly advancing, and ID clinicians should plan now for LLMs or other AI tools to soon mimic expert-level functionality across a range of ID consultations and with a degree of consistency that healthcare system stakeholders deem acceptable. We therefore implore the ID community to familiarize themselves with these tools, including their potential benefits and limitations, to enable informed participation in urgent conversations about the future role of AI in healthcare. Now more than ever, ID physicians must unequivocally establish the irreplaceable value of human cognitive specialists in patient care.

## Supplementary Data

Supplementary materials are available at *Clinical Infectious Diseases* online. Consisting of data provided by the authors to benefit the reader, the posted materials are not copyedited and are the sole responsibility of the authors, so questions or comments should be addressed to the corresponding author.

## Supplementary Material

ciad633_Supplementary_Data

## Notes

***Financial support*.** None.
