# Supplementary material for: Black Box Warning: Large Language Models and the Future of Infectious Diseases Consultation
Source: Clin Infect Dis. 2023 Nov 16;78(4):860–6. doi: 10.1093/cid/ciad633 (PMC11006107; doi:10.1093/cid/ciad633)
Supplement: ciad633_Supplementary_Data [file ciad633_supplementary_data.docx]

**Box S1**. Dangerous confabulations in a management plan for HIV-associated disseminated histoplasmosis created by ChatGPT (GPT-3.5) and verified by ChatGPT (GPT-4).

| Clinician | please write a detailed management plan for a patient with HIV-associated disseminated histoplasmosis. Please include references to support recommendations |
| --- | --- |
| ChatGPT (GPT-3.5) | **Diagnosis and Initial Management:**  The diagnosis of disseminated histoplasmosis in a patient with HIV should be based on clinical and laboratory findings. The most common presenting symptoms are fever, fatigue, weight loss, and respiratory symptoms. Laboratory tests should include blood cultures, serum and urine antigen tests, and histopathological examination of tissues or bone marrow aspirates. Once the diagnosis is confirmed, treatment should be started immediately.  **Antifungal Therapy:**  The first-line therapy for disseminated histoplasmosis in HIV-infected patients is liposomal amphotericin B (L-AmB) 3-5 mg/kg/day for 2 weeks, followed by oral itraconazole 200 mg three times daily for 12 months. In patients with severe disease, high fungal burden, or in those who are unable to tolerate L-AmB, a higher dose of 7.5-10 mg/kg/day can be considered. After initial treatment with L-AmB, it is essential to monitor renal function and electrolytes regularly.  Voriconazole or posaconazole can be used as an alternative to itraconazole in patients with intolerance or contraindications to itraconazole. For patients who are unable to tolerate azoles due to adverse effects or drug interactions, intravenous echinocandins such as caspofungin or anidulafungin may be used as an alternative. In refractory cases, combination antifungal therapy with two or more agents may be considered.  **Management of Immune Reconstitution Syndrome (IRS):**  HIV-infected patients with disseminated histoplasmosis may develop IRS after the initiation of antiretroviral therapy (ART). This can occur due to the restoration of immune function and the subsequent inflammatory response to residual Histoplasma antigens. IRS may manifest as fever, worsening respiratory symptoms, lymphadenopathy, or new lesions. Treatment of IRS includes the continuation of antifungal therapy and the initiation of corticosteroids at a dose of prednisone 1 mg/kg/day for 2-4 weeks, followed by a gradual taper.  **Secondary Prophylaxis**:  After completing the induction phase of antifungal therapy, secondary prophylaxis with itraconazole 200 mg once daily or posaconazole 400 mg once daily should be continued for life or until CD4 count is ≥ 150 cells/mm3 for at least 6 months with sustained HIV viral suppression. In patients unable to tolerate or have contraindications to azoles, primary prophylaxis with L-AmB 3-5 mg/kg once weekly can be considered.  **Management of Complications:**  Complications of disseminated histoplasmosis include respiratory failure, disseminated intravascular coagulation (DIC), and adrenal insufficiency. In patients with respiratory failure, mechanical ventilation may be required. In patients with DIC, supportive measures including transfusion of blood products and anticoagulation may be required. Adrenal insufficiency should be managed with hydrocortisone replacement therapy.  In conclusion, disseminated histoplasmosis is a severe opportunistic infection in HIV-infected patients that requires early diagnosis and prompt initiation of antifungal therapy. Itraconazole is the first-line therapy, and L-AmB is reserved for severe cases. Corticosteroids may be necessary for the management of IRS, and secondary prophylaxis should be continued indefinitely. Close monitoring for complications is essential for improving patient outcomes.  References:  Wheat LJ, Freifeld AG, Kleiman MB, et al. Clinical Practice Guidelines for the Managementof Patients with Histoplasmosis: 2007 Update by the Infectious Diseases Society of America. Clin Infect Dis. 2007;45(7):807-825. doi:10.1086/520818  Wheat J, Myint T, Guo Y, et al. Central nervous system histoplasmosis: Multicenter retrospective study on clinical features, diagnostic approach and outcome of treatment. Medicine (Baltimore). 2018;97(37):e12572. doi:10.1097/MD.0000000000012572  Perfect JR, Dismukes WE, Dromer F, et al. Clinical practice guidelines for the management of cryptococcal disease: 2010 update by the Infectious Diseases Society of America. Clin Infect Dis. 2010;50(3):291-322. doi:10.1086/649858 |
| Clinician | what is the evidence to support echinocandins in this disease? |
| GPT-3.5 | The use of echinocandins in disseminated histoplasmosis remains controversial, and there is limited clinical evidence to support their use. Although some in vitro and animal studies have shown activity against Histoplasma species, clinical data on their efficacy in human infections are limited.  A few case reports and case series have suggested that echinocandins may have a role in the treatment of disseminated histoplasmosis, particularly in patients who are intolerant or refractory to azole therapy. In a case series of three patients with disseminated histoplasmosis who were refractory to amphotericin B and itraconazole, caspofungin was added to the regimen, resulting in a favorable outcome in all three cases [1]. Another case report described the successful use of micafungin in a patient with disseminated histoplasmosis who had failed prior therapy with amphotericin B, itraconazole, and posaconazole [2].  A retrospective study of 36 patients with disseminated histoplasmosis who received caspofungin as salvage therapy reported a response rate of 64% and a 6-month survival rate of 44% [3]. However, this study had several limitations, including its retrospective nature, small sample size, and the inclusion of patients with different underlying conditions, making it difficult to draw definitive conclusions about the role of echinocandins in the treatment of disseminated histoplasmosis.  Overall, the use of echinocandins in disseminated histoplasmosis should be considered on a case-by-case basis, and their use should be reserved for patients who are intolerant or refractory to azole therapy or amphotericin B. Further studies are needed to better define the role of echinocandins in the management of this disease.  References:  1. Santos RP, Martínez-Gamboa A, Reyes-Montes Mdel R, et al. Successful treatment of refractory disseminated histoplasmosis with caspofungin in three patients with different immune profiles. Ann Clin Microbiol Antimicrob. 2007;6:4. doi:10.1186/1476-0711-6-4  2. Kanthula RM, Uppin SG, Pamidi U, et al. Micafungin therapy for disseminated histoplasmosis. J Glob Infect Dis. 2013;5(1):28-31. doi:10.4103/0974-777X.106393  3. Caceres DH, Zuluaga A, Arango-Bustamante K, et al. Echinocandins as Salvage Therapy for Refractory Disseminated Histoplasmosis: A Case Series and Review of the Literature. Open Forum Infect Dis. 2019;6(2):ofz030. doi:10.1093/ofid/ofz030 |
| Clinician | Please review this management plan for HIV associated disseminated histoplasmosis for accuracy: [the above output was pasted in a new chat] |
| ChatGPT (GPT-4) | Your management plan for HIV-associated disseminated histoplasmosis is generally accurate and comprehensive. However, a few points should be addressed:   1. The correct term for IRS is "Immune Reconstitution Inflammatory Syndrome (IRIS)," not "Immune Reconstitution Syndrome." 2. The dose of prednisone for managing IRIS should be specified as 1 mg/kg/day up to 60 mg/day, not just 1 mg/kg/day. 3. The source you cited regarding primary prophylaxis with L-AmB should be updated. Please consider using more recent guidelines from the Infectious Diseases Society of America (IDSA) for updated information on prophylaxis recommendations.   Other than these points, your management plan is well-organized and includes relevant information on diagnosis, antifungal therapy, management of IRIS, secondary prophylaxis, and management of complications.  It is essential to keep up-to-date with the latest guidelines and research, as management recommendations may change over time. Always consult the most recent guidelines and evidence-based resources for accurate information. |
| Comment | There are several serious errors here. Firstly, the liposomal amphotericin B dose for severe disease (which includes virtually all patients with HIV-associated disseminated histoplasmosis) was incorrectly stated as 7.5-10 mg/kg/day (the initially mentioned dose of 3-5 mg/kg/day was correct). Secondly, the dose of itraconazole is incorrect (should be 200 mg PO three times daily for 3 days, then 200 mg PO twice daily), which could lead to serious toxicities. Thirdly, the suggestion that echinocandins can be used as an alternative to amphotericin B and azoles is dangerously wrong (echinocandins do not have activity against Histoplasma in vivo). When asked in a follow-up question about the evidence to support echinocandins in histoplasmosis, GPT-3.5 confabulates a 36-patient case series that does not exist.  The initial management plan was provided in a new chat to Chat GPT (GPT-4) for cross-checking. It does detect incorrect term “immune reconstitution syndrome” but fails to catch the much more serious medication errors. It also incorrectly asks for more recent IDSA guidelines. Note the “falsehood mimicry” in the GPT-4 parrots the erroneous term, “primary prophylaxis” when referring to secondary prophylaxis. |

**Box S2**. Full transcript of dangerous confabulations that could be overlooked by a non-specialist, when ChatGPT is asked for a management plan for HIV-associated cryptococcal meningitis.

| Clinician | Please write a detailed management plan for a patient with cryptococcal meningitis. Include references. |
| --- | --- |
| ChatGPT (GPT-3.5) | Cryptococcal meningitis is a serious infection caused by the fungus Cryptococcus neoformans. It primarily affects individuals with compromised immune systems, such as those with HIV/AIDS or who have received organ transplants. Prompt diagnosis and management are crucial to improve outcomes and prevent mortality. Here is a detailed management plan for a patient with cryptococcal meningitis:  1. Diagnosis: The diagnosis of cryptococcal meningitis is confirmed by analyzing cerebrospinal fluid (CSF) obtained by lumbar puncture. The presence of Cryptococcus neoformans antigen or the fungus itself on CSF examination confirms the diagnosis.  2. Antifungal therapy: The cornerstone of management is antifungal therapy, which aims to eliminate the fungus from the CSF and prevent recurrence. The induction phase involves the use of amphotericin B, in combination with flucytosine, for 2 weeks. Subsequently, patients are transitioned to fluconazole, given at high doses for an additional 8-10 weeks, followed by maintenance therapy with lower doses for up to 1 year. The choice and duration of therapy may vary depending on patient factors, such as severity of illness and immune status.  3. Management of raised intracranial pressure: Cryptococcal meningitis can cause increased intracranial pressure (ICP), which can lead to neurological complications. In patients with elevated ICP, prompt management is essential, including administration of acetazolamide, mannitol or hypertonic saline, and consideration of CSF drainage if necessary.  4. Immune reconstitution: Immune reconstitution therapy with antiretroviral therapy (ART) should be initiated in patients with HIV/AIDS as soon as possible, to improve immune function and prevent recurrence of cryptococcal meningitis. ART should be initiated within 2 weeks of antifungal therapy initiation, provided there are no contraindications.  5. Management of complications: Patients with cryptococcal meningitis may develop various complications, including hydrocephalus, seizures, and focal neurological deficits. These should be promptly recognized and managed with appropriate interventions, such as ventriculoperitoneal shunting, antiepileptic therapy, and rehabilitation.  6. Follow-up: Close follow-up is necessary to monitor for response to therapy and identify potential complications. Serial lumbar punctures may be necessary to monitor for clearance of fungus from the CSF. ART should be continued in patients with HIV/AIDS, and regular monitoring of immune function and viral load should be performed.  7. Prevention: Cryptococcal meningitis can be prevented in patients with HIV/AIDS by early initiation of ART, and prophylaxis with fluconazole may be considered in high-risk patients. Transplant recipients may require prophylaxis with antifungal agents for a prolonged duration.  References:  1. Perfect JR, Dismukes WE, Dromer F, et al. Clinical practice guidelines for the management of cryptococcal disease: 2010 update by the infectious diseases society of America. Clin Infect Dis. 2010;50(3):291-322.  2. Sloan DJ, Parris V. Cryptococcal meningitis: epidemiology and therapeutic options. Clin Epidemiol. 2014;6:169-182.  3. World Health Organization. Guidelines for the diagnosis, prevention and management of cryptococcal disease in HIV-infected adults, adolescents and children. Geneva, Switzerland: World Health Organization; 2018. |
| Clinician | Please assess this management plan for cryptococcal meningitis: [the above output was pasted in a new chat] |
| ChatGPT (GPT-4) | This management plan for cryptococcal meningitis appears comprehensive and well-informed, adhering to clinical practice guidelines and recommendations from reputable sources. The plan outlines diagnostic methods, antifungal therapy, management of increased intracranial pressure, immune reconstitution, management of complications, follow-up, and prevention. It takes into account the unique challenges of treating cryptococcal meningitis in HIV/AIDS patients and organ transplant recipients, emphasizing early initiation of antiretroviral therapy (ART) and prophylaxis in high-risk populations.  The sources cited at the end of the plan further support its credibility, including the 2010 update of the clinical practice guidelines for cryptococcal disease management by the Infectious Diseases Society of America, a review article on cryptococcal meningitis epidemiology and therapeutic options, and the World Health Organization's guidelines for diagnosing, preventing, and managing cryptococcal disease in HIV-infected individuals.  Overall, the management plan seems well-rounded and evidence-based, addressing the primary aspects of cryptococcal meningitis diagnosis, treatment, and prevention. It is essential to tailor this plan to individual patients, considering their specific clinical presentations and needs. |
| Clinician | When should ART be started? please provide references |
| GPT-4 | According to the guidelines for the diagnosis, prevention, and management of cryptococcal disease in HIV-infected adults, adolescents, and children, antiretroviral therapy (ART) should be initiated within 2 weeks of starting antifungal therapy for cryptococcal meningitis, provided there are no contraindications. This recommendation is based on evidence suggesting that earlier initiation of ART improves immune function and reduces the risk of mortality and recurrence of cryptococcal meningitis.  Reference:  World Health Organization. Guidelines for the diagnosis, prevention and management of cryptococcal disease in HIV-infected adults, adolescents and children. Geneva, Switzerland: World Health Organization; 2018. Available from: https://www.who.int/hiv/pub/guidelines/cryptococcal-disease/en/  It is important to closely monitor patients for signs of immune reconstitution inflammatory syndrome (IRIS) when initiating ART, as this can lead to a paradoxical worsening of symptoms. Management of IRIS may require corticosteroids and other immunomodulatory therapies. |
| Clinician | what about the RCT by boulware et al in NEJM 2010 that showed higher mortality when ART was started within 2 weeks? |
| GPT-4 | You are right to bring up the study by Boulware et al., published in the New England Journal of Medicine (NEJM) in 2010. In this randomized controlled trial (RCT), the researchers found that starting antiretroviral therapy (ART) within 2 weeks of initiating treatment for cryptococcal meningitis was associated with higher mortality, compared to starting ART after 2 weeks.  Reference: Boulware DR, Meya DB, Muzoora C, et al. Timing of antiretroviral therapy after diagnosis of cryptococcal meningitis. N Engl J Med. 2014;370(26):2487-2498. doi:10.1056/NEJMoa1312884  It is important to weigh the potential risks and benefits when deciding when to start ART in the context of cryptococcal meningitis. The RCT by Boulware et al. demonstrates that there may be risks associated |
